# Does grapiprant reduce osteoarthritic pain in dogs?

**DOI:** 10.18849/ve.v11i2.732

**Published:** 2026-04-14

**Authors:** Grace Olding, Merran Govendir

**Affiliations:** The University of Sydney Australia

**Keywords:** CANINE, DOG, EP-4 ANTAGONIST, GRAPIPRANT, OSTEOARTHRITIS

## Abstract

**PICO question:**

In dogs with osteoarthritis, does grapiprant, compared with no grapiprant, reduce osteoarthritic pain?

**Clinical bottom line:**

**Category of research:**

Treatment.

**Number and type of study designs reviewed:**

Two randomised controlled trials.

**Strength of evidence:**

Weak.

**Outcomes reported:**

One study found that dogs with osteoarthritis given grapiprant were found to have significant clinical improvement in both owner assessed outcomes (treatment successes, pain interference scores, pain severity scores) and veterinarian assessed outcomes (total orthopaedic scores) when compared to a placebo. The second study of dogs with acute induced arthritis found no significant differences in vertical force ratios or veterinarian assessed visual lameness scores between dogs administered grapiprant and the control group.

**Conclusion:**

These studies suggest that grapiprant reduces the impact of osteoarthritic pain on activities of daily living and orthopaedic scores in dogs but does not improve lameness associated with acute, severe osteoarthritic pain. Given the limited and inconsistent evidence, further appropriate studies are required to determine the efficacy of grapiprant in managing osteoarthritic pain in dogs.

How to apply this evidence in practice

The application of evidence into practice should take into account multiple factors, not limited to: individual clinical expertise, patient’s circumstances and owners’ values, country, location or clinic where you work, the individual case in front of you, the availability of therapies and resources.

Knowledge Summaries are a resource to help reinforce or inform decision making. They do not override the responsibility or judgement of the practitioner to do what is best for the animal in their care.

## Clinical scenario

A 12-year-old, spayed Labrador Retriever presents with mild osteoarthritic pain. The dog has been treated with firocoxib with satisfactory results; however, it has had to be discontinued based on azotaemia and elevated Symmetric dimethylarginine (SDMA), evidencing kidney pathology. The veterinarian is now considering the use of grapiprant because of its safety profile, however, is uncertain whether its efficacy is supported by clinical evidence.

## The evidence

Two randomised controlled studies were found to match the PICO question (Rausch-Derra et al., (2016); de Salazar Alcalá et al., (2019)). The first study, Rausch-Derra et al. (2016), found that grapiprant was significantly more effective than a placebo based on both the owner’s assessment and the veterinarian’s assessment in client-owned dogs with osteoarthritis assessed over 28 days. The de Salazar Alcalá et al. (2019) trial found grapiprant was not significantly more effective than no grapiprant, as measured by vertical force ratios and visual lameness scores in dogs with induced synovitis assessed over 24 hours.

These studies provide weak evidence that grapiprant, compared to no grapiprant, reduces osteoarthritic pain in dogs with osteoarthritis. In addition to their conflicting findings, both studies have significant limitations. Additional appropriate studies are required, and the decision to use grapiprant remains dependent on the clinician’s judgement.

## Summary of the evidence

**Rausch‐Derra et al. (2016):** A Prospective, Randomized, Masked, Placebo‐Controlled Multisite Clinical Study of Grapiprant, an EP4 Prostaglandin Receptor Antagonist (PRA), in Dogs with Osteoarthritis **Aim: **To evaluate the efficacy of grapiprant compared to a placebo in dogs with osteoarthritis.

Population:	Client-owned, otherwise healthy dogs from 16 hospitals across the United States with confirmed osteoarthritis, aged 0.5 to 16.75 years and weighing from 4.1 to 70.4 kg.
Sample size:	262 dogs.
Intervention details:	Dogs were randomly assigned to 1 of 2 treatments (grapiprant, n = 131; placebo, n = 131). 11 dogs were withdrawn for treatment Owners administered grapiprant (2 mg/kg) per OS (PO) or placebo PO daily for 28 days. Owners completed the Canine Brief Pain Inventory (CBPI) on day 0 (baseline), days 7, 14, 21, and 28. Veterinarians completed physical examinations and the veterinary assessment of the total orthopaedic score (TOS) at screening (baseline) and on days 14 and 28.
Study design:	Prospective, multicentre, double-blinded, randomised, placebo-controlled, clinical trial, parallel study.
Outcome Studied:	Owner assessment using the CBPI to calculate a pain severity score (PSS) and the pain interference score (PIS) on a numerical scale, and an overall categorical impression of the dog’s quality of life over the last 7 days. The primary effectiveness variable was defined as the CBPI score on day 28 compared to the CBPI score at day 0.Veterinarian assessment of the most severely affected appendicular joint, excluding intervertebral joints, was made to assign each dog a total orthopaedic score (TOS).
Main Findings(relevant to PICO question):	Treatment successes were significantly higher in the grapiprant treatment group compared to the placebo treatment group (P = 0.0315). The pain interference score (PIS) and pain severity score (PSS) improved significantly in the grapiprant treatment group compared to placebo treatment group (P = 0.0029 and P = 0.0022, respectively). TOS were significantly better in the grapiprant treatment group compared to the placebo treatment group (P = 0.0086).
Limitations:	The population was heterogeneous; while age and weight were covariates in the analysis, other factors such as breed, body condition score and severity of osteoarthritis were potentially confounding factors. There was no mention of lifestyle factors, such as diet (except nutraceutical diets) or exercise, that could impact outcomes, nor measures to control them. Dogs were not required to be fasted when dosed, which may have affected the absorption of grapiprant. The hundreds of owners and large number of assessors across various facilities introduced variability. Adverse gastrointestinal signs (vomiting, diarrhoea, inappetence) could have had the effect of unmasking the owners to the fact that their dog was receiving the drug rather than placebo. The study was funded by Aratana Therapeutics, in support of the Food and Drug Administration (FDA) approval of grapiprant. Three of the four authors were employed by Aratana Therapeutics and the fourth was a paid consultant.

**de Salazar Alcalá et al. (2019):** Assessment of the efficacy of firocoxib (Previcox®) and grapiprant (Galliprant®) in an induced model of acute arthritis in dogs **Aim: **To compare the efficacy of firocoxib and grapiprant with a control in dogs with induced acute arthritis.

Population:	Healthy Beagle dogs from a research facility in France with no history of lameness or gait abnormality, aged 12 to 41.5 months and weighing from 8.7 to 13.5 kg.
Sample size:	18 dogs (12 dogs pertaining to PICO question).
Intervention details:	This study compared three treatment groups (control, firocoxib, grapiprant). Only the treatment groups pertaining to the PICO question are discussed below (control, grapiprant). The dogs were ranged by weight and randomly allocated to the three groups (Group 1 control, n = 6; Group 3 grapiprant, n = 6). Two experiments were conducted with each group with a 26 day wash out in between. Dogs in Group 1 (controls) were untreated (i.e. no placebo administered). Dogs in Group 3 (grapiprant) were administered a single dose of grapiprant per os (PO) at a dose rate of 1.5 to 2.9 mg/kg for each of the two experiments. Dogs were fasted for at least 12 h before treatments. Dogs were anaesthetised 2 and 14 h after the dose in experiments 1 and 2, respectively, with propofol (6.5 mg/kg as an intravenous injection). Transient osteoarthritis was induced by an intraarticular injection of a sodium urate crystal suspension into the femorotibial joint (experiment 1 – right joint; experiment 2 – left joint). For each assessment, a dog was repeatedly walked across the force plate to obtain three interpretable hind-limb values. Experiment 1: Outcomes were assessed at 1.5, 3, 5, 7, and 10 h post induction of arthritis (3.5 h to 12 h after dose of grapiprant). Experiment 2: Outcomes were assessed at 1.5, 3, 5, 7, and 10 h post induction of arthritis (15.5 h to 24 h after dose of grapiprant).
Study design:	A randomised, two-sequence, assessor-blinded study involving two separate experiments.
Outcome Studied:	Mean force-plate derived lameness (vertical force) ratios were compared between groups at defined times post-arthritis induction. The ratio between the mean force applied across the force-plates after treatment and the mean baseline force of the same hind limb measured two days before arthritis induction (the ‘lameness ratio’ = ‘vertical force ratio’), was the primary variable chosen to assess pain control.Visual lameness scores observed by one blinded operator at each time point were used as secondary criteria of assessment.
Main Findings(relevant to PICO question):	In both experiment 1 and experiment 2, no significant differences in vertical force ratios were observed between Group 3 (grapiprant) and Group 1 (controls). In both experiment 1 and experiment 2, there was no significant difference in mean visual lameness scores between the Group 3 (grapiprant) and Group 1 (controls) at each time point post-arthritic induction.
Limitations:	The short study duration of 24 hours and single dose of grapiprant does not encapsulate the intended usage of grapiprant and may not reflect the outcomes of this use. A large range of doses (5 to 2.9 mg/kg) was used. It is unclear whether these doses were normalised over the weight of each dog. The induced synovitis model of acute arthritis assesses acute pain control which may not reflect the outcomes of chronic pain control in degenerative osteoarthritis. The observed lameness may be attributable to the volumetric effect of the intra-articular injection, resulting in capsular distension or altered joint laxity. The dose-response to the urate crystals varies between individuals and different studies (one failed induction in this study) and the pain caused by the urate crystals is not constant over time. Experiment 1 and 2 could not be compared due to the 26-day interval and different joints used for urate crystal injections. The force plate gait analysis (FPGA) has the limitation of only evaluating the dog one specific time point, and measures weight bearing which is only one component of chronic pain in osteoarthritic dogs. The small sample size undermines the internal and external validity the study and could reflect a large effect size, which may be problematic if grapiprant is only indicated for mild to moderate pain. No power analysis was provided to justify the small sample size and subsequent statistical analysis. It is unclear whether the study will detect clinically relevant differences as no basis for the effect size is provided through literature or a pilot study. The study was funded by Merial, the manufacturer of Previcox (firocoxib). One author was the sponsor of the study and an employee of the pharmaceutical company. This sponsor prepared the study’s design, was involved in monitoring, reporting and analysing results and writing the publication drafts.

## Appraisal, application and reflection

Osteoarthritis is a chronic, progressive and painful synovial joint disease, prevalent in geriatric dogs (Pye et al., 2022). Effective pain management is critical to maintaining quality of life in this population (Pye et al., 2022). Non-steroidal anti-inflammatory drugs (NSAIDs) are generally accepted as the first-line treatment for canine osteoarthritis (Pye et al., 2022). While these prostanoids mediate pain and inflammation, they also have constitutive actions in maintaining the gastric mucosa, renal function, and platelet aggregation (de Salazar Alcalá et al., 2019; Pye et al., 2022). However, conventional NSAIDs inhibit the cyclooxygenase (COX) pathway and thus the prostanoid molecules that cascade from it. This means that their inhibition is also associated with adverse effects, including gastrointestinal, renal, and hepatic toxicity (de Salazar Alcalá et al., 2019; Pye et al., 2022).

Prostaglandin E2 (PGE2) is the most significant prostaglandin the synovium and the primary mediator of canine osteoarthritis pain and inflammation (Gruen et al., 2022; Rausch-Derra et al., 2015; Ricciotti & FitzGerald, 2011). Prostaglandin E2 receptor 4 (EP4) is the primary receptor for PGE2 (de Salazar Alcalá et al., 2019). Grapiprant, a prostaglandin receptor antagonist, is a relatively novel therapeutic agent in the management of canine osteoarthritis that selectively blocks the EP4 receptor rather than the entire COX pathway (de Salazar Alcalá et al., 2019; Nagahisa & Okumura, 2016). As a more targeted therapy, grapiprant has demonstrated a favourable safety profile and has therefore been proposed as a possible alternative treatment in dogs who are unable to tolerate COX-inhibiting NSAIDs, (Gruen et al., 2022; Rausch-Derra et al., 2015).

A caveat to this safety profile is the recent finding that grapiprant is a P-glycoprotein (P-gp) substrate (Mealey et al., 2022). P-glycoprotein is a drug transporter and therefore plays an important role in drug disposition (Mealey et al., 2022). P-glycoprotein substrates have the potential to cause life-threatening adverse drug reactions resulting from impaired P-gp function in dogs with the P-gp gene or acquired from drug-drug interactions (Mealey et al., 2022).

Rausch-Derra et al. (2016) and de Salazar Alcalá et al. (2019) are the only studies to date comparing the efficacy of grapiprant with a control group in managing osteoarthritic pain in dogs. These studies have vastly different study designs and report conflicting findings regarding the effectiveness of grapiprant in treating canine osteoarthritic pain.

The study population in the Rausch-Derra et al. (2016) trial consisted of a heterogeneous population of dogs with osteoarthritis of varying age, breed, and sex in a field setting. This heterogeneity introduced potentially confounding factors, including body condition, breed, and severity of osteoarthritis (age and weight were covariates in the analysis). Additionally, there was no mention of lifestyle factors that may affect outcomes, such as diet (aside from nutraceutical diets) or exercise, nor of any measures implemented to control them. This is a limitation, as both dietary management and exercise are first-line recommendations for osteoarthritis management (Gruen et al., 2022). Maintenance of a lean body condition reduces mechanical strain on joints and slows cartilage degradation, while exercise can reduce pain and inflammation through modulation of opioid, serotonin, and dopamine pathways and improve limb function (Impellizeri et al., 2000; Kealy et al., 2000; Marshall et al., 2010; Rychel, 2010; Runhaar et al., 2015; Frye et al., 2016; Lima et al., 2017). Ideally, participants should have been assessed for these factors and instructed to maintain them consistently throughout the study. Furthermore, unlike the subjects in de Salazar Alcalá et al. (2019), dogs in the Rausch‐Derra et al. (2016) trial were not required to be fasted prior to dosing. This is problematic because grapiprant absorption is optimised in a fasted state (111.9% bioavailability in a fasted state compared to 59.1% in a non-fasted state), although the time at which concentrations exceed the minimal effective concentration is unaffected (Łebkowska-Wieruszewska et al., 2016). Likewise, the adverse gastrointestinal effects observed in dogs during the study (vomiting, diarrhoea, inappetence) may have further affected drug absorption and excretion. The de Salazar Alcalá et al. (2019) study population was significantly more homogenous, consisting of healthy research Beagles in a laboratory setting, although it was markedly younger than the general osteoarthritis population. However, unlike the Rausch-Derra et al. (2016) trial, which had explicit and well‐defined criteria, the screening and enrolment criteria were vague, making scrutiny and replication difficult.

The duration of the Rausch-Derra et al. (2016) trial was 28 days, which the authors cited as a potential limitation. The half-life of grapiprant is about 6 hours, and when administered every 24 hours the accumulation is 1.07 (Łebkowska-Wieruszewska et al., 2016). Therefore, drug accumulation is unlikely to be an issue with this dosing regimen, and 28 days should be sufficiently long to detect potential improvement. This is consistent with current labelling guidelines, which recommend discontinuing use if no clinical improvement is apparent after 14 days (Elanco, 2023). The 24 hour duration of the de Salazar Alcalá et al. (2019) trial is more problematic. A single dose may be insufficient to elicit a clinical response, which, according to current labelling guidelines, is usually observed within 7 days, and the short duration increases susceptibility to transient fluctuations in pain (Elanco, 2023). As a drug commonly prescribed as a daily baseline medication for osteoarthritis, a longer study length would have been preferable.

Outcome assessment in Rausch-Derra et al. (2016) involved multiple assessors, which may have inter-observer variability and reduced measurement accuracy and reduced accuracy. In contrast, the use of a single assessor in de Salazar Alcalá et al. (2019) may reduce the reliability of the measurements. Both studies involved outcomes assessed in a veterinary setting. Anxiety can increase pain reactivity, pain sensitivity, or both (Caddiell et al., 2023). Neither study reported strategies to reduce patient stress, such as low-stress handling or environmental modifications (Gruen et al., 2022).

There are also blinding issues in both studies. In de Salazar Alcalá et al. (2019), assessors of the outcomes were blinded but those administering the treatment were not. While different personnel were used, it is unclear whether an effective firewall between these sets of personnel was maintained. A double-blinded trial with a placebo or blind assessment of outcome by independent personnel in a non-blinded trial would have been preferable. In Rausch-Derra et al. (2016), although double-blinded, adverse gastrointestinal signs, such as vomiting, diarrhoea, and inappetence, may have unmasked the owners to the fact that their dog was receiving the drug rather than placebo. It may have been advantageous to assess this by asking participants during or after the trial whether they thought they were receiving grapiprant or the placebo to ensure their responses were random (Shmerling, 2022).

The Canine Brief Pain Inventory (CBPI) used in the Rausch-Derra et al. (2016) study is validated to measure chronic pain associated with canine osteoarthritis and to detect treatment response (Brown et al., 2007; Brown et al., 2008). The CBPI has the advantage of integrating observations over both an extended period and ‘right now’, which accommodates the waxing and waning nature of the disease (Brown et al., 2008). Furthermore, it is completed in the home setting where the dog is likely to be most relaxed, and by an individual most familiar with its behaviour (Brown et al., 2013b). The owner assessment is, however, a subjective outcome measure (Brown et al., 2013a). While the study states that the format and content of the CBPI were presented to the owner in the validated form, the time between the screening appointment and the start of the study ranged from 1 to 7 days. It is recommended to begin baseline data collection at least 7 to 10 days after the screening appointment to minimise regression to the mean associated with owners seeking out a study when their dog's clinical severity is exacerbated (Brown et al., 2013b). For the veterinary assessment, only a single joint was assessed, which could underestimate or overestimate the effect of grapiprant, although any bias would be equally likely in both treatment groups.

The force plate gait analysis (FPGA) in the de Salazar Alcalá et al. (2019) study is the current best method for evaluating lameness. Under appropriately controlled conditions, FPGA has the advantage of being an objective measure that is highly reliable and sensitive to change. However, weight bearing on an affected limb is only one component of chronic pain in osteoarthritic dogs (Brown et al., 2007). Furthermore, FGPA has the limitation that it evaluates the dog at a single time point, outside its normal environment, which may restrict the generalisability of the results (Brown et al., 2013a).

In de Salazar Alcalá et al. (2019), the dogs were ranged by weight and randomly allocated into the three groups in blocks of three. The lameness ratios and visual lameness scores were analysed using analysis of the variance with repeated measurements on time and a significance level of α = 0.05. The statistical analysis section is scant in detail, making it difficult to scrutinise or repeat. For example, the study does not report whether the data were normally distributed or whether the large range of doses (1.5 to 2.9 mg/kg) was normalised for the weight of each dog. Moreover, a similar study found the responses of individual dogs, as in Rausch-Derra et al. (2016), a better method for evaluating treatment effects than lameness scores (Brown et al., 2013b).

Both studies have significant conflicts of interest, which may undermine the validity and reliability of the results (Dickersin & Chalmers, 2011; Lundh et al., 2017; Naci et al., 2014). Both studies were funded by pharmaceutical companies, with no firewall separating the sponsor of the study and the evaluation of the drug. The Rausch-Derra et al. (2016) study was funded by the pharmaceutical company Aratana Therapeutics in support of the Food and Drug Administration (FDA) approval of grapiprant. Three of the four authors were employed by Aratana Therapeutics, and the fourth was a paid consultant for Aratana Therapeutics. Likewise, the de Salazar Alcalá et al. (2019) study was funded by Merial, the manufacturer of Previcox (firocoxib). While the study was conducted by an independent organisation, one of the four authors was the sponsor of the study and employee of the pharmaceutical company. This sponsor prepared the study’s design, was involved in monitoring, reporting and analysing results and wrote the publication drafts. While such associations with corporations are commonplace, a Cochrane review found that industry sponsored trials had a 1.27 times higher risk of reporting favourable efficacy results and 1.34 times higher risk of reporting favourable overall conclusions than non-industry sponsored trials (Lundh et al., 2017). These biases cannot be explained by standard ‘risk of bias’ assessment tools (Lundh et al., 2017).

Notably, these studies assess different aspects of pain, complicating direct comparisons between the studies. While the CBPI quantifies the impact of pain on activities of daily living, the FPGA quantiﬁes the impact of pain on lameness. CBPI pain scores and vertical force measures have been shown to have ‘no correlation or concordance’ in efficacy trials for osteoarthritis, suggesting that they quantify diﬀerent aspects of chronic pain (Brown et al., 2013). In reconciling these results, it may be that grapiprant reduces the impact of chronic pain on activities of daily living in dogs with osteoarthritis but does not improve lameness. This, however, may be an oversimplification, given that the veterinary orthopaedic scores were also improved in Rausch-Derra et al. (2016). These included a lameness component and an examination of weight-bearing, range of motion, pain or resistance on palpation, and swelling.

Given that there are only two studies relevant to this PICO question, and that these report conflicting findings, it is helpful to interpret these studies within the context of all published papers that evaluate the efficacy of grapiprant in treating joint pain in dogs. This review yields three additional papers; Budsberg et al. (2019), Cassemiche et al. (2024), and Enomoto et al. (2024).

Budsberg et al. (2019) conducted a blinded, three-way crossover study using five purpose-bred hound-cross dogs in which synovitis was experimentally induced via an intra-articular injection of sodium urate. The study measured vertical ground reaction forces and subjective clinical lameness evaluations during standing, walking, and trotting (scored on a scale of 0 to 11) at baseline (0 hours) and at 6, 12, 24, 36, and 48 hours post-injection. Each dog received four doses of three treatments – L-766 (a proprietary EP4 receptor antagonist, 4.0 mg/kg), grapiprant (EP4 antagonist, 2.0 mg/kg), and carprofen (4.4 mg/kg) – administered 14 and 2 hours before and 22 and 46 hours after the sodium urate injection. Of the three treatments, grapiprant was the least effective in reducing vertical ground reaction forces and subjective clinical lameness scores caused by induced synovitis.

A more recent study by Cassemiche et al. (2024) employed a randomised, double-blinded, prospective clinical trial involving 54 client-owned dogs with naturally occurring unilateral cranial cruciate ligament rupture treated via tibial plateau levelling osteotomy (TPLO). Starting the day after surgery, all dogs received a subcutaneous dose of meloxicam (0.2 mg/kg) and then were randomly assigned 1:1 to receive either oral grapiprant (2 mg/kg) or meloxicam (0.1 mg/kg) once daily for 14 days. Pain severity and pain interference scores assessed using the CBPI were conducted on days 3, 7, 10, and 15 postsurgery, and veterinary orthopaedic scores assessed on day 15 post-surgery. Three days after surgery, grapiprant treated dogs had lower PSS compared to meloxicam treated dogs. PIS was also significantly lower in the grapiprant group at day 3 and at day 10.

A third study, Enomoto et al. (2024), was an open-label study of 48 young dogs (mean ± SD age of 30.7 ± 10.7 months) with osteoarthritis, obvious joint pain, and a Liverpool Osteoarthritis in Dogs (LOAD) score of ≥ 5. The dogs were given multimodal treatment of grapiprant at the recommended dose daily, omega-3 fatty acid supplementation was initiated at 100 mg/kg and then increased to 200 mg/kg daily, and leash exercise was gradually increased to a target of 60 minutes daily. Client-reported outcome measures and force plate gait analysis were collected at baseline and monthly for 4 months, indexed to the most severely affected limb at baseline. Client-reported outcome measures showed significant improvement over time and at each time point. Overall, peak vertical force increased significantly, and vertical impulse increased numerically.

Budsberg et al. (2019) and Cassemiche et al. (2024) reinforce the pattern that the reported efficacy of grapiprant correlates with the outcome studied. Cassemiche et al. (2024) observed this correlation even when the intervention was postsurgical articular pain rather than osteoarthritic pain. While Enomoto et al. (2024) has the benefit of combining force-plate analysis and client-reported outcomes, the open-label, multimodal study design without a control group limits interpretability, particularly as exercise alone is known to reduce osteoarthritic joint pain (Rychel, 2010; Runhaar et al., 2015; Frye et al., 2016; Lima et al., 2017).

Pain sensitivity and emotional reactivity are complex and individual to each animal, and the ability to perceive and ascribe emotions, including pain, to another being is a feature of empathy (Gruen et al., 2020). This makes cross-species identification of pain particularly fallible (Caddiell et al., 2023). Taken together, Rausch-Derra et al. (2016), Budsberg et al. (2019), de Salazar Alcalá et al. (2019), Cassemiche et al. (2024) and Enomoto et al. (2024) provide weak evidence that grapiprant, compared to no grapiprant, reduces osteoarthritic pain in dogs. Additional appropriate studies are required.

## Methodology

**Search Strategy:** 

Databases searched and dates covered:	CAB Abstracts (1910—2024) via Web of SciencePubMed (1966—2024) via National Center for Biotechnology Information
Search strategy:	CAB Abstracts:(TS=(osteoarthritis) OR TS=(osteo-arthritis) OR TS=(arthritis) OR TS=(joint disease) OR TS=(joint diseases) OR TS=(DJD) OR TS=(pain) OR TS=(chronic pain)) AND (TS=(dog) OR TS=(dogs) OR TS=(canine) OR TS=(canines) OR TS=(canis) OR TS=(canid) OR TS=(canids) OR TS=(Canidae)) AND (TS=(galliprant) OR TS=(grapiprant) OR TS=(piprant) OR TS=(prostaglandin receptor antagonist) OR TS=(EP4 receptor antagonist))PubMed:((osteoarthritis) OR (osteo-arthritis) OR (arthritis) OR (joint disease) OR (joint diseases) OR (DJD) OR pain OR (chronic pain)) AND ((dog) OR (dogs) OR (canine) OR (canines) OR (canis) OR (canid) OR (canids) OR (Canidae)) AND ((galliprant) OR (grapiprant) OR (piprant) OR (prostaglandin receptor antagonist) OR (EP4 receptor antagonist))
Dates searches performed:	3 June 2025

**Exclusion / Inclusion Criteria:** 

Exclusion:	Systematic reviews, narrative reviews, in vitro studies, conference papers, and book chapters.
Inclusion:	Papers relevant to the PICO question, and randomised controlled trials.

**Search Outcome:** 

Database	Number of results	Excluded – narrative review	Excluded – in vitro study	Excluded – randomised control but not relevant to PICO question	Total relevant papers
CAB Abstracts	27	10	0	15	2
PubMed	37	14	4	16	3
Total relevant papers when duplicates removed					2

## Acknowledgements

This work was completed in partial fulfillment for the requirements of the Doctor of Veterinary Medicine degree, The University of Sydney. This research was (in part) funded by the Sydney School of Veterinary Science Research and Enquiry Unit of Study 2024 fund.

## Author contributions

**Grace Olding:**Writing - Original Draft, Writing - Review & Editing. **Merran Govendir:**Writing - Review & Editing, Supervision.

## ORCiD

Grace Olding: https://orcid.org/0000-0001-6360-6825 Merran Govendir: https://orcid.org/0000-0003-3595-0500

## Conflict of Interest

The authors declare no conflicts of interest.

## Disclaimer

Knowledge Summaries are a peer-reviewed article type which aims to answer a clinical question based on the best available current evidence. It does not override the responsibility of the practitioner. Informed decisions should be made by considering such factors as individual clinical expertise and judgement along with patient’s circumstances and owners’ values. Knowledge Summaries are a resource to help inform and any opinions expressed within the Knowledge Summaries are the author's own and do not necessarily reflect the view of the RCVS Knowledge. Authors are responsible for the accuracy of the content. While the Editor and Publisher believe that all content herein are in accord with current recommendations and practice at the time of publication, they accept no legal responsibility for any errors or omissions, and make no warranty, express or implied, with respect to material contained within. For further information please refer to our Terms of Use.
